# Implantable Systems for Continuous Liquorpheresis and CSF Replacement

**DOI:** 10.7759/cureus.1022

**Published:** 2017-02-10

**Authors:** Manuel Menéndez González

**Affiliations:** 1 Neurology, Hospital Universitario Central de Asturias

**Keywords:** csf, shunt, filtration, neurodegenerative, neuroimmunology, alzheimer, parkinson, guillain-barre, device, ventriculo-peritoneal shunt

## Abstract

Liquorpheresis (cerebrospinal fluid filtration) comprises a therapeutical approach that has been proposed to treat several neurological conditions where antibodies, inflammatory mediators, or abnormal peptides are the cause or play an important role in the pathogenesis of the disease. Continuous or intermittent cerebrospinal fluid (CSF) replacement may be an alternative approach not explored thus far.

Here, we review previous experiences in the use of liquorpheresis in autoimmune and degenerative neurological diseases. Then we describe previous technical reports  and provide some new innovations in order to design bidirectional CSF shunting systems that can be complemented either with a deposit of artificial CSF or with a filter of CSF, allowing CSF replacement or liquorpheresis respectively. Both options would lead to mechanical dilution of the patient’s CSF.

## Introduction

Liquorpheresis (CSF filtration) comprises a new therapeutical approach that has been proposed to treat several neurological conditions where soluble proteins or peptides within the nervous system play an important role in the physiopathology of the disease. This is the case of several autoimmune diseases where antibodies are involved affecting the central nervous system or the nerve roots. Among them, Guillain-Barrè syndrome (GBS) is perhaps the one with more evidence supporting its use. This disorder is usually treated with infusion of intravenous immunoglobulins. Corticoids, immunosuppressants and plasma exchange are alternatives. A clinical trial tested liquorpheresis in GBS. Although the number of patients was small, it was found that liquorpheresis is at least as effective as with plasma exchange [1, 2]. Multiple Sclerosis (MS), other autoimmune disorder, is usually treated with immunomodulatory and immunosuppressant drugs. Plasmapheresis was used with some success in open and controlled trials in some cases with acute or fulminant course of the disease. Probably, a better option than plasmapheresis would be liquorpheresis since the autoimmune reaction is produced within the central nervous system. In a pilot-study where ten MS patients were treated with liquorpheresis, six patients improved in some extent. In all MS patients CSF-filtration was well tolerated, adverse events or complications were not observed [3]. Other autoimmune diseases where liquorpheresis was tried are single case reports and therefore provide limited evidence. For instance, in a case of  lupus erythematosus with cerebral involvement liquorpheresis led to rapid and lasting neurological improvement and clinical cure [4].

The production and deposit of toxic peptides is a central fact in the physiopathology of all neurodegenerative diseases. The distinct protein aggregates that are found in neurodegenerative disorders and prion diseases seem to cause these disorders. Small intermediates (soluble oligomers) in the aggregation process can confer synaptic dysfunction, whereas large, insoluble deposits can cause cellular damage. Findings in other neurodegenerative diseases indicate that a broadly similar process of neuronal dysfunction is induced by diffusible oligomers of misfolded proteins [5]. Then, we propose that neurodegenerative diseases are also suitable to be treated by removing those proteins from CSF. For instance Alzheimer’s disease (AD) might be treated by removing beta-amyloid and phospho-Tau, Parkinson’s disease (PD) and Lewy Bodies disease might be treated by removing alpha-synuclein, Huntington’s disease (HD) by removing huntingtin protein and so on. Indeed, liquorpheresis has already been tried in some neurodegenerative diseases, such as in one patient with familial amyotrophic lateral sclerosis (ALS), where it did not show positive results [6].

Even when liquorpheresis might represent a good therapeutic option in many neurological conditions, it has not been explored intensively yet, probably because it is not widely available. We devise bidirectional CSF derivation systems allowing portable liquorpheresis. Alternatively, CSF can be replaced with artificial cerebrospinal fluid (aCSF). Both options would lead to mechanical dilution of the patient’s CSF.

## Technical report

The following paragraphs have the objective of describing previous developments and provide some new technical innovations in order to design implantable systems for continuous liquorpheresis and CSF replacement.

### State of the art

Standard Ventriculoperitoneal Derivation

Ventricular-peritoneal derivation  is a method of diverting CSF from the ventricles to the abdominal cavity. It is the usual treatment of hydrocephalus, a condition produced when the natural system for draining and absorbing CSF does not work properly and the ventricles enlarge to accommodate the extra fluid. Systems for ventricular-peritoneal derivation allow draining CSF from the ventricular system (or from the spinal subarachnoid space in the case of lumbar-peritoneal derivation) to the peritoneum. A derivation usually consists of two simple catheters (single light) and a one-way valve. The valve regulates the amount of CSF going out of the brain ventricles. As pressure increases within the brain cerebrospinal fluid, the check valve opens and excess liquid is drained to another body. There are various types of derivations. The two most common are: 1. Fixed pressure valves that regulate the amount of cerebrospinal fluid is drained based on a predetermined pressure setting. 2.  Adjustable pressure valves that regulate the amount of cerebrospinal fluid that is drained based on a pressure value which can be adjusted. Using specially designed magnetic tools, neurosurgeons may change noninvasively the pressure level of the implanted adjustable valve during a visit in consultation, without another surgical procedure. Both types of valve may include an anti-siphon device control. The purpose of an anti-siphon device control is minimize overdrainage due to the force of gravity, which can cause more cerebrospinal fluid to drain when the individual is upright. In addition, some valves include a reservoir that can be used to check the operation of the derivation and to obtain samples of cerebrospinal fluid for laboratory studies.

Lumboperitoneal Derivations and Implantable Intrathecal Pumps

Lumboperitoneal shunting is also a method of diverting CSF to the abdominal cavity, but this one diverts CSF from the lumbar subarachnoid space instead of from the ventricles. Advantages of lumboperitoneal derivations over ventricular-peritoneal derivations include avoidance of brain penetration with the shunt catheter, access to a large CSF space in the thecal sac, and the potential of a large amount of CSF drainage. Patients who require a lumboperitoneal shunt have pathology that predisposes to obstruction of CSF absorption or elevated CSF pressure but must have communication of the spinal CSF with the cranial compartment.

Implantable Intrathecal pumps are medical devices used to deliver medications directly into the lumbar subarachnoid space. They consist of a small battery-powered, programmable pump that is implanted under the subcutaneous tissue of the abdomen and connected to a small catheter tunneled to the site of spinal entry. Sophisticated drug dose regimens can be instituted. Implanted pumps need to be refilled every few months.

Bidirectional Ventriculoperitoneal Derivation

Patent US20090131850A1, entitled “Method and apparatus for removing harmful proteins from a mammalian's ventricular cerebrospinal fluid” devices bidirectional ventricular-peritoneal derivations complemented with a pump and a filter allowing the removal of proteins from CSF [7]. Then, it can be considered the first claimed patent claim of an implantable system for continuous liquorpheresis.

Artificial CSF (aCSF) has been first produced in 1949 and refined since then with the purpose of using it as a vehicle solution for administration of agents to the central nervous system and neuroendoscopic surgery [8-9]. Yang Qin and Jian W. Gu have proposed a bidirectional ventricular-peritoneal  derivation with a pump infusing aCSF to improve the homeostasis of CSF, as a new treatment for AD, very recently [10].

### Description of implantable systems for liquorpheresis and CSF replacement

General Description

**Figure 1:**
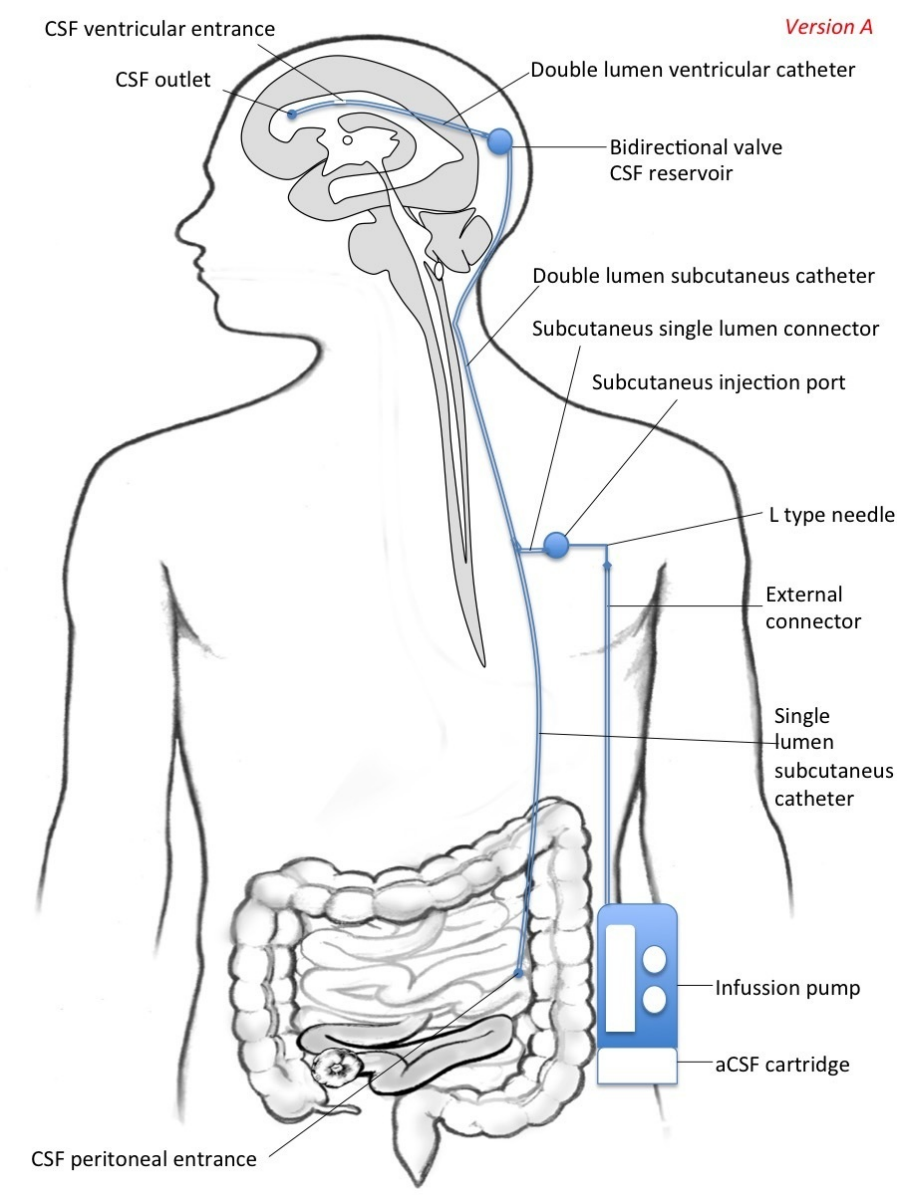
Version A, Model A: A Portable System for CSF Replacement from the Ventricular System The fluid to be infused is externally administered to the pump with a cartridge. The subcutaneous simple drainage catheter drains through an orifice into the peritoneal space (alternatively, it could be collected in an external collector).

**Figure 2:**
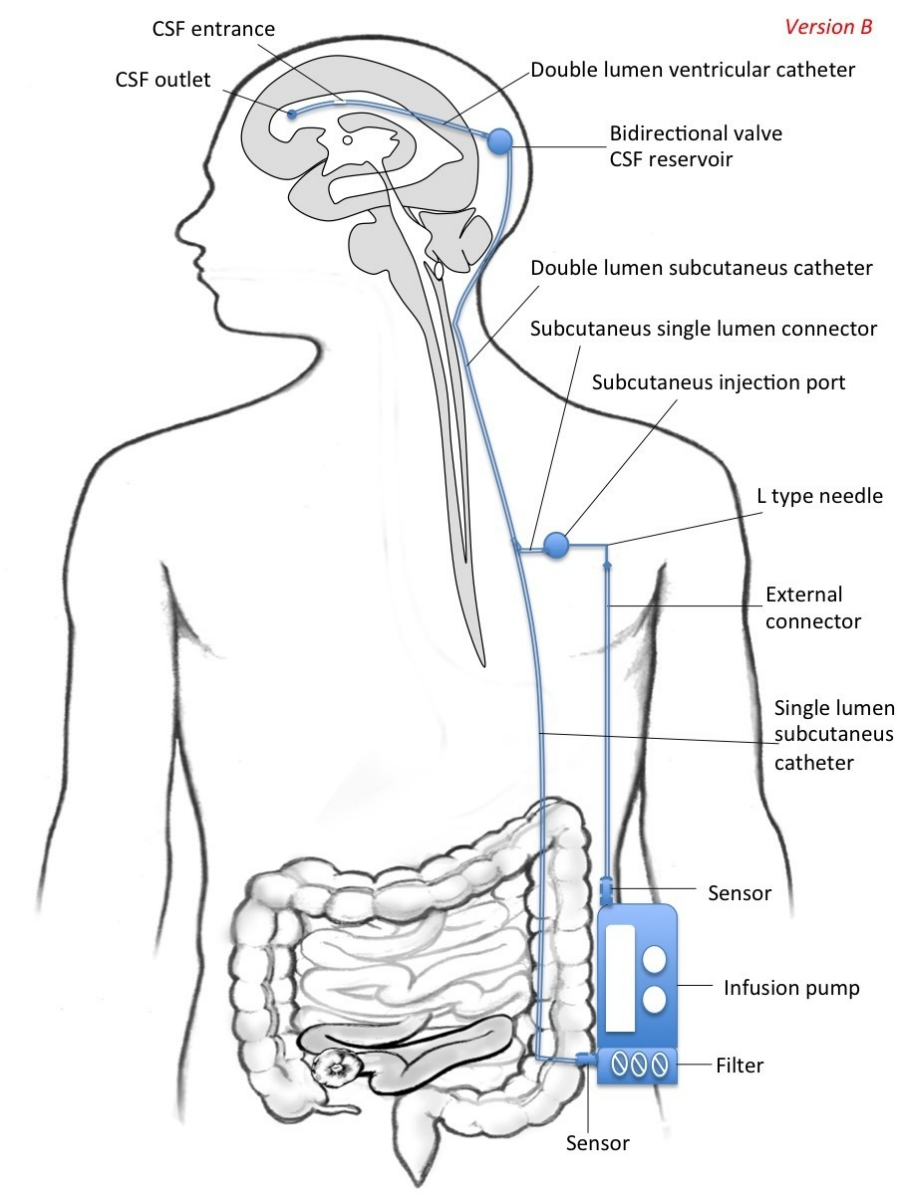
Version B, Model A: A Portable System for Liquorpheresis from the Ventricular System There is a filter of CSF attached to the pump, which takes its source from the single subcutaneous drainage catheter. Also, the filter/pump device may be endowed with a sensor in the entry and exit connectors.

Standard ventricular-peritoneal  (or lumbar-peritoneal) derivations does not allow diluting CSF as catheters are unidirectional. Using current technology as starting point, we envision new developments to construct implantable systems aimed at infusing either aCSF (CSF replacement) or the own patient’s CSF after filtration (liquorpheresis). This model comprises a series of interconnected elements, reservoirs, and access ports, allowing both to drain CSF to the outside (to an external collector) or to the peritoneum and to infuse CSF into the ventricular system (ventriculo-peritoneal) or the spinal subarachnoid space (lumbo-peritoneal derivation). Infusing CSF can be both aCSF (CSF replacement) and filtered CSF (liquorpheresis). Key elements in this model are the following (figure 1 and figure 2):

- Double lumen ventricular catheter  (one inlet and one output), which allows both draining CSF from the ventricular system and infusing fluids. As an alternative to placement in the ventricular system (ventriculo-peritoneal derivation), the catheter can also be placed at the spinal level in the lumbar subarachnoid space (lumbo-peritoneal derivation).

- Double valve (bi-directional), which allows to regulate the pressure both input and output.

- Double lumen subcutaneous catheter  (one inlet and one output, connected to homonyms ventricular catheters through the double valve).

- A subcutaneous injection port connected to the gateway subcutaneous catheter, which allows fluid infusion by an external pump. Additionally it can also allow access for infusing drugs with syringe if needed.  

- External pump allowing continuous or intermittent infusion. It is connected to the injection port by an infusion apparatus with an L type non-coring needle.

Versions and Models

This system may be constructed in two different versions intended for a) CSF replacement (version A)  and b) CSF filtration (version B). Thus, the pump can be complemented with a deposit of aCSF (version A) or with a filter (version B). Additionally, the system may be intended for ventricular shunting (model A) or for intrathecal shunting (model B).

In version A (Figure 1), the fluid to be infused is externally administered to the pump from a cartridge. The subcutaneous simple drainage catheter drains through an orifice into the peritoneal space (or outside). Thus, the proximal and the distal ends of the draining catheter are placed into the frontal horn of the lateral ventricle (or in the spinal subarachnoid space) and the peritoneal cavity (or to an external collector), respectively. aCSF has been first produced in 1949 and refined since then with the purpose of using it as a vehicle solution for administration of test agents to the central nervous system and neuroendoscopic surgery [7-8]. Today, with the availability of aCSF, CSF replacement seems a feasible intervention from a technical point of view. Up to our knowledge, continuous or intermittent CSF replacement has not been explored so far in the treatment of neurological conditions.

In version B (Figure 2) , there is a filter of CSF attached to the pump, which takes its source from the single subcutaneous drainage catheter. Thus, the proximal and the distal ends of the draining  catheter are placed into the frontal horn of the lateral ventricle (or in the spinal subarachnoid space) and to the filter, respectively. Some filters of CSF have already been developed and patented, but no one small enough to be used in a portable device. Several different types of filters can be developed in the future; from mechanical filters, similar to those used in plasmapheresis, to "biological filters", where antibodies against the different proteins catch them from the CSF (Figure 4). Biological filters of CSF may represent a highly innovative field of research combining nanotechnology with immunotechnology (ie magnetic nanoparticles conjugated with antibodies). Finally, the filter-pump device may be endowed with sensors in the entry and exit connectors, in order to measure the levels of proteins in the CSF entering and leaving the pump (Figure 2).

**Figure 3:**
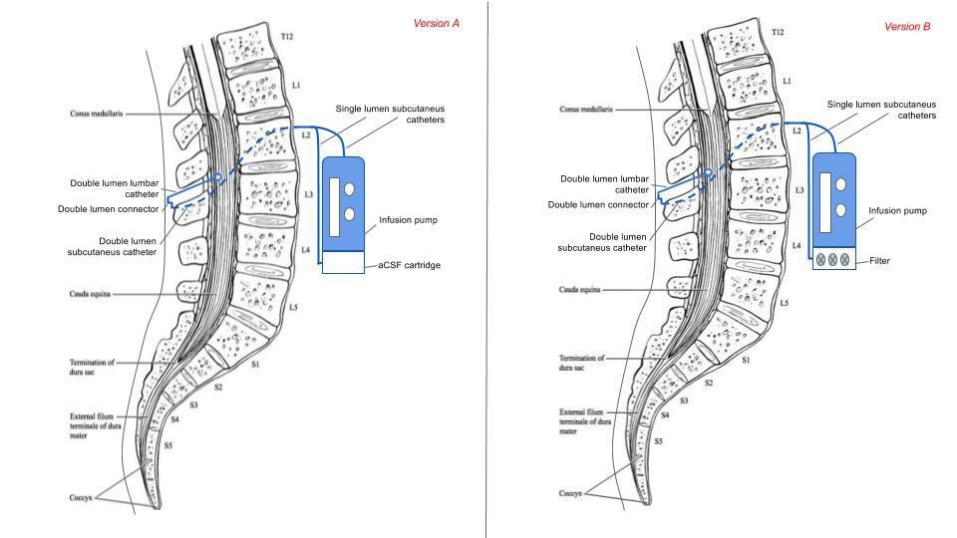
Portable Systems for CSF Replacement and Liquorpheresis from the Intrathecal Space *Version A, Model B (on the left): *a portable system for CSF replacement from the intrathecal space. A lumbar catheter takes out CSF that is collected in an external collector (alternatively it could be drained into the peritoneal space). The fluid to be infused is externally administered to the pump with a cartridge. *Version B, model B (on the right)*: a portable system for liquorpheresis from the intrathecal space. There is an LCR filter attached to the pump, which takes its source from the single subcutaneous drainage catheter.

Basically, the difference between model A (ventricular shunting, figure 1 and figure 2) and model B (intrathecal shunting, figure 3) is the point where the central catheter is placed: in the lateral ventricles or in the lumbar subarachnoid space respectively. Then, the techniques for implanting these systems would be similar to those used to implant ventricular-peritoneal derivations for model A and lumbar-peritoneal derivations or implantable intrathecal pumps for model B. Both models allow the implementation of versions A and B.

The system by Qin & Gu, and patent  conceives an external pump that can be feed with deposits of aCSF [10]. However, pumps used today to deliver intrathecal drugs are implanted in the subcutaneous tissue and are refilled using syringes and needles. Thus, CSF replacement could be addressed with implantable pumps that can be refilled with aCSF. The same way, if long-duration filters were available, implanting them with the pump in the subcutaneous tissue would be a good option for liquorpheresis. Then, both versions A and B (and models A and B), can also be conceived as totally implantable systems with no external components.

**Figure 4:**
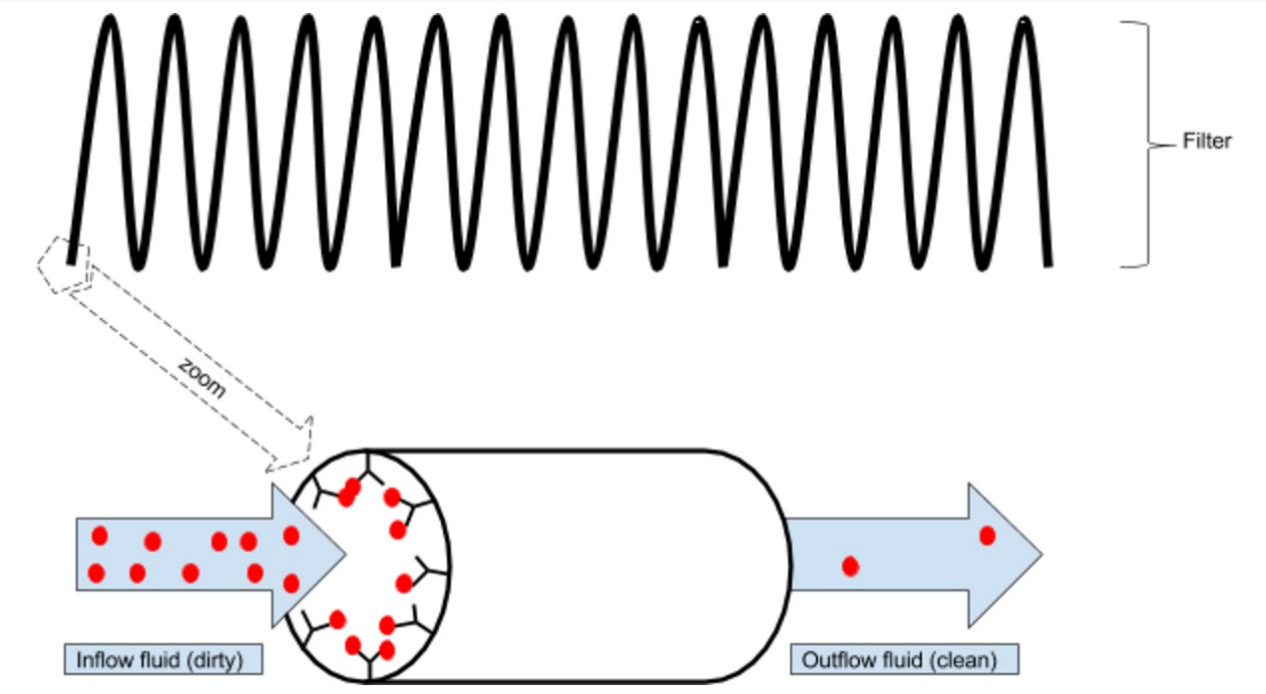
Schematic representation of a filter based on immunotechnology The inside of a long column is fully coated with antibodies against the target molecule (red balls). As the fluid passes through the filter, the target molecules get stuck to the antibodies. As a result, the concentration of target molecules is much lower in the outflow fluid than in the inflow fluid.

## Discussion

Today, with the availability of aCSF, CSF replacement seems a feasible intervention from a technical point of view. Up to our knowledge, CSF replacement has not been explored  in the treatment of neurological conditions so far, although it has recently been proposed to treat AD by Qin&Gu [10]. They propose to use a bidirectional ventricular-peritoneal derivation to replace CSF with aCSF.

The most innovative element in the systems described in this paper, is that the pump can be complemented not only with a deposit of aCSF (as in the system by Qin&Gu) but alternatively it can be complemented with a filter (as in patent US20090131850A1), allowing CSF replacement or liquorpheresis respectively. Both options ease the dilution of  the “dirty” CSF (with neurotoxic proteins) with “clean” CSF (without neurotoxic proteins) which can help fighting many neurological conditions.

In order to maximize the dilutional effect, the system may be programed to work in an alternate mode where the drainage and infusion of clean CSF is sequentially produced to avoid the new and “clean” CSF mixing with all the “dirty” CSF in the ventricular system that would happen if the system worked in  a continuous in-out mode. Thus, some amount of “dirty” CSF should be drained before the infusion of “clean” CSF starts.

The best way of programming the system has to be studied carefully to maximize the clearance effect and reduce the risk of side effects to a minimum. Side effects may appear as a consequence of producing liquoral hypotension (if too much “dirty” CSF is drained) or hypertension (if too much “clean” CSF is infused) in a given time. However, well-adjusted valves should prevent this type of malfunctioning.

Other types of side effects may be related to filters and aCSF. Filters should be as specific as possible, removing only the target peptides and proteins. aCSF should have the same composition as natural CSF [11].  As the systems here described are closed systems (only version A has the option of draining into the peritoneum), infections are expected to be less frequent than in ventriculo-peritoneal and lumbo-peritoneal derivations. Anyway, the management of infections would be exactly the same, with the advantage that in these models antiseptics can be infused using the pump.

## Conclusions

Liquorpheresis (CSF filtration) and replacement of CSF with aCSF comprise new therapeutical approaches that have been proposed to treat several neurological conditions. Both options have been little explored so far due to technical constraints.

Developing implantable systems for liquorpheresis and CSF replacement seems suitable today from a technical point of view. We describe previous technical reports  and provide some new innovations in order to design bidirectional CSF shunting systems that can be complemented either with a deposit of artificial CSF or with a filter of CSF, allowing CSF replacement or liquorpheresis respectively. Both options would lead to mechanical dilution of the patient’s CSF.
